# Ovarian stimulation and oocyte cryopreservation in females and transgender males aged 18 years or less: a systematic review

**DOI:** 10.3389/fendo.2023.1146476

**Published:** 2023-06-19

**Authors:** Marnie Slonim, Michelle Peate, Kira Merigan, Daniel Lantsberg, Richard A. Anderson, Kate Stern, Debra Gook, Yasmin Jayasinghe

**Affiliations:** ^1^ Oncofertility Program and Department of Gynaecology, Royal Children’s Hospital, Melbourne, VIC, Australia; ^2^ Department of Obstetrics and Gynaecology, Royal Women's Hospital, The University of Melbourne, Melbourne, VIC, Australia; ^3^ Department of Obstetrics and Gynaecology, Royal Women’s Hospital, Melbourne, VIC, Australia; ^4^ Melbourne IVF, Melbourne, VIC, Australia; ^5^ MRC Centre for Reproductive Health, University of Edinburgh, Edinburgh, United Kingdom

**Keywords:** oocyte cryopreservation, fertility preservation, ovarian hyperstimulation, ovarian stimulation (OS), paediatric and adolescent gynaecology, oocyteretrieval, oncofertility, paediatric oncofertility

## Abstract

**Background:**

Fertility preservation is an important healthcare focus in the paediatric and adolescent population when gonadotoxic treatments are required. Ovarian stimulation (OS) resulting in oocyte cryopreservation is a well-established fertility preservation option in the adult population. It’s utility, however, is little known in young patients. The purpose of this review was to synthesise the available literature on OS in patients ≤18 years old, to identify gaps in current research and provide suggestions for future research directions.

**Methods:**

Using PRISMA guidelines, a systematic review of the literature was performed for all relevant full-text articles published in English in Medline, Embase, the Cochrane Library and Google Scholar databases. The search strategy used a combination of subject headings and generic terms related to the study topic and population. Two reviewers independently screened studies for eligibility, extracted data and assessed the risk of bias. Characteristics of the studies, objectives and key findings were extracted and summarised in a narrative synthesis.

**Results:**

Database search and manual review identified 922 studies, 899 were eliminated based on defined exclusion criteria. Twenty-three studies were included and comprised 468 participants aged ≤18 years who underwent OS (median 15.2, range 7-18 years old). Only three patients were premenarchal, and four patients were on treatment to suppress puberty. Patients had OS for a broad range of indications including oncology treatment, transgender care and Turner syndrome. A total of 488 cycles of OS were completed, with all but 18 of these cycles (96.3%) successfully resulting in cryopreserved mature oocytes (median 10 oocytes, range 0-35). Fifty-three cycles (9.8%) were cancelled. Complications were rare (<1%). One pregnancy was reported from a female who had OS aged 17 years old.

**Conclusion:**

This systematic review demonstrates that OS and oocyte cryopreservation is achievable in young females however there are only a few cases in the literature describing OS in premenarcheal children or those who have suppressed puberty. There is little proof that OS can lead to pregnancy in adolescents, and no proof that this can be achieved in premenarchal girls. Therefore it should be regarded as an innovative procedure for adolescents and experimental for premenarcheal girls.

**Systematic review registration:**

https://www.crd.york.ac.uk/prospero/display_record.php?RecordID=265705, identifier CRD42021265705.

## 1 Introduction

Fertility preservation is now an important component of healthcare in the paediatric and adolescent population where treatment involves risk to future fertility, most commonly because of administration of gonadotoxic agents (1). Therapies for cancer, rheumatological or haematological diseases, and for gender dysphoria, may be detrimental to the ovary at any age (2). Similarly, a range of genetic conditions, most prominently Turner syndrome (TS), may result in premature ovarian insufficiency at an early age. Future infertility is a significant source of concern and anxiety for both a young patient and their family members in these circumstances (1).

Oncofertility services are developing rapidly around the globe to support those at risk of treatment-related infertility and assist with fertility preservation in a timely manner (3). Therapies to protect or restore fertility are well established in the adult female population (2, 4, 5); however, data and options are limited in the paediatric and adolescent population. Clinicians may find it challenging to discuss and offer invasive fertility preservation treatments to young people with little data on proven long-term benefit (6, 7).

For many years clinicians have used ovarian shielding, transposition away from the radiation field, and GnRH analogues in an attempt to protect fertility, which have conflicting or scarce evidence of benefit, particularly in minors (8, 9). More modern fertility preservation options include ovarian tissue cryopreservation (OTC), *in vitro* maturation (IVM) and ovarian stimulation (OS) for oocyte cryopreservation (2, 10).

Until very recently ovarian tissue cryopreservation has been the only assisted reproduction technology (ART) offered for pre-pubertal girls and post-pubertal females where there is limited time before cancer treatment (11). It is considered an established procedure in adult women with around 200 births reported to date, but so far, there have been only 2 live births from premenarcheal tissue (12, 13). IVM involves retrieval of immature oocytes from ovaries after minimal or no gonadotrophin stimulation and their subsequent maturation in the laboratory. In the context of fertility preservation, collection of immature oocytes from adult ovarian tissue and IVM is experimental and very few livebirths have been reported (14). OS resulting in oocyte or embryo cryopreservation is the most successful form of fertility preservation for biological females (15), however, it has been studied mainly in adult populations (16). Additionally, there are questions around oocyte quality in very young women, as studies of follicle morphology have demonstrated an increase in abnormal types in the young (17). Embryo cryopreservation poses ethical issues in the young and may prove limiting in the event of partner change (18).

Given that many patients will only have one opportunity to preserve fertility prior to commencing gonadotoxic treatment, it is important that they are offered preservation options that will give them the greatest chance to achieve future parenthood. There are reasons why oocyte cryopreservation may be considered an addition to, or preferred to OTC in selected populations. A single stimulation cycle followed by a minimally invasive oocyte retrieval, compared with laparoscopy and its associated recovery for OTC, may make the procedure more acceptable to some patients (19). The possibility of reintroducing malignant cells in patients diagnosed with haematological cancers (3, 20) means that reimplantation of untreated ovarian tissue may not be considered safe in some cases. In patients with genetic conditions with increased risk of premature ovarian insufficiency where the pathology is intrinsic to the ovary, such as TS, the accelerated germ cell loss with thawing and ovarian transplantation has led to uncertainty about the likely success of ovarian tissue reimplantation (21).

The aim of this systematic review is to evaluate oocyte cryopreservation, by means of OS in the paediatric and adolescent population. We compare age, diagnosis and pubertal and menarchal status and comment on success rates, adverse outcomes, and psychological morbidity. Additionally, we identify future research directions that may support the successful adoption of these therapies around the world.

## 2 Methods

### 2.1 Search strategy

In accordance with the Preferred Reporting Items for Systematic Reviews and Meta-Analyses (PRISMA) guidelines, a systematic search of the literature was performed for all relevant full-text articles published in English in Medline, Embase, the Cochrane Library, and Google Scholar databases (PROSPERO registration number CRD42021265705).

The following search terms were used in different combinations: “ovarian stimulation”, “oocyte cryopreservation”, “*in vitro* fertilization (IVF)”, “fertility preservation” (see **Supplementary Material** for all the search terms and search strategy). A final search was conducted on 14/08/2022 to ensure inclusion of all relevant studies.

### 2.2 Study selection

Articles were included if they reported on any clinical outcomes of oocyte cryopreservation in the paediatric and adolescent (≤18 years old) population. Studies that included patients with other fertility preservation procedures were included if data for the individual subtypes of fertility preservation procedures were reported separately. Studies that only described alternative fertility preservation options or reported on outcomes in those >18 years old were excluded.

Case series, prospective and retrospective comparative cohorts, controlled (non-randomised) and randomised controlled trials, review articles, cross-sectional studies, and case reports were included. Guidelines, commentaries, conference abstracts, and pilot study data that were also reported in a published study already included in the review were excluded.

**Figure 1:**
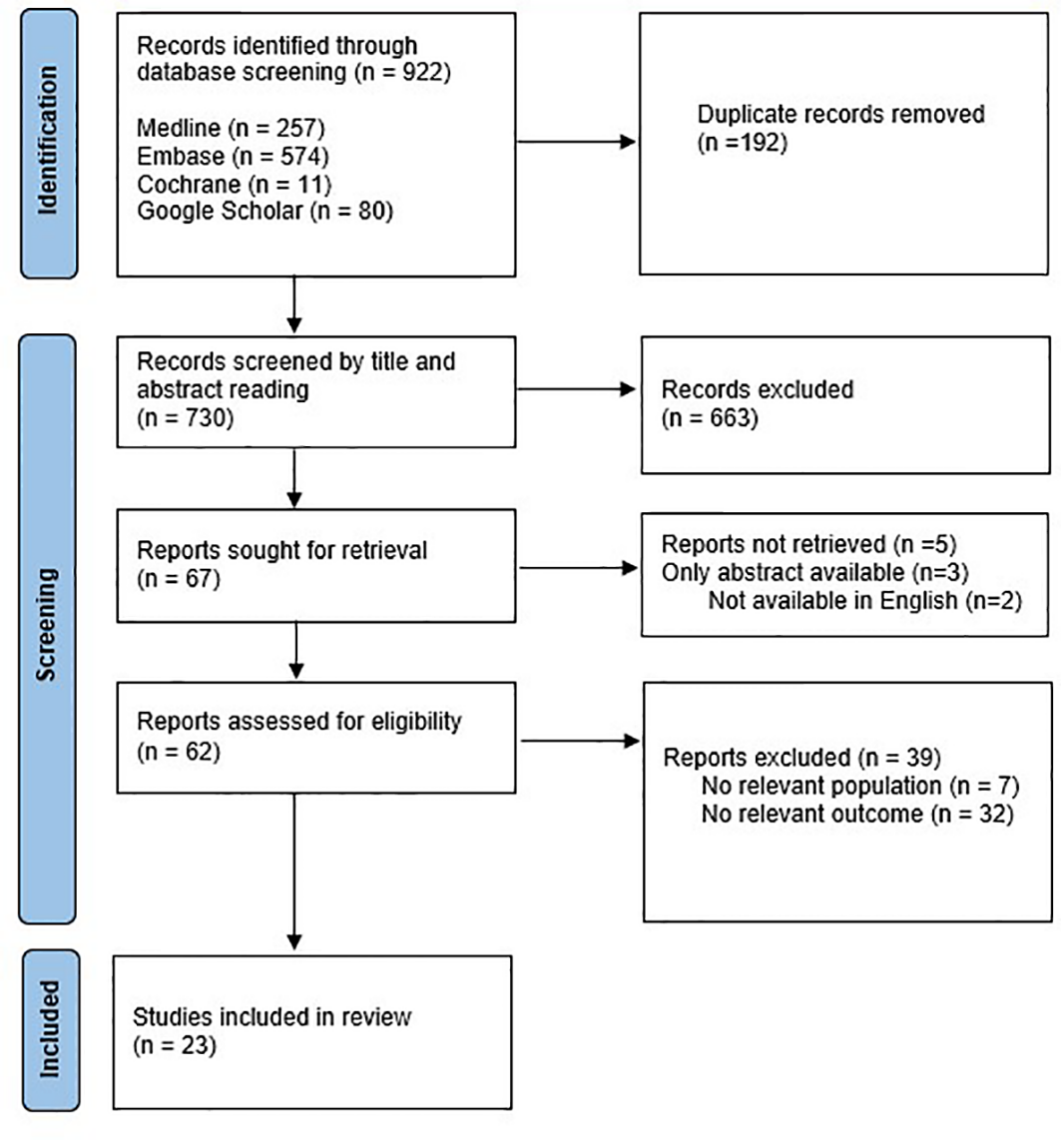
Identification of studies *via* databases and registers (22).

References (n=922) were imported into a Covidence database where duplicates were removed. The remaining abstracts (n = 730) were subsequently reviewed independently by two authors (MS, KM) and all those describing outcomes of COS or oocyte cryopreservation in females 18 years or younger underwent full text review. Based on title and abstract screening, 663 articles were excluded, 67 full text articles were assessed for eligibility and as 5 articles were not accessible, 62 were eligible for review (**Figure 1**).

### 2.3 Data extraction and analysis

Data from articles were extracted into a pre-designed database. Year of publication, country of study, study objectives, study design, sample size, patient characteristics, intervention, outcome measures, and findings were documented. No relevant outcomes were found for 32 articles and 7 articles did not discuss the relevant population and were therefore excluded. The remaining 23 studies were included for systematic review. No discrepancies were found, therefore a third reviewer was not required for definitive decisions on the data extraction.

The two independent reviewers performed methodological quality assessment for each study. Due to the range of study designs being analysed, Qualsyst (Appendices 1, 2) was used to facilitate the assessment of risk of bias for each study. Each study received a percentage score. Any discrepancy was resolved through discussion.

We attempted to correspond with some study investigators to resolve data queries and request additional data as required regarding undocumented pubertal or menarchal status, side effects to treatments, or sub analysis of age groups, and included relevant additional information provided.

## 3 Results

### 3.1 Study characteristics

The 23 papers reviewed were from USA (n=16), UK (n=3), Israel (n=2), Sweden (n=1), and China (n=1). They include case reports (n=10), case series (n=6), retrospective cohort studies (n=4), prospective cohort studies (n=2), and a letter to the editor describing a case report (n=1) (**Table 1**) (10, 19, 23–43).

**Table 1:** Description of studies examining age, pubertal and menarchal status, diagnosis, ovarian reserve testing, and oocytes retrieved and cryopreserved (see **Supplementary Material** for more detailed results in table format).

Author, Country	Diagnosis	Study design	Age (years) median (range)	Pubertal status	Ovarian reserve testing: median (range)^#^			Controlled ovarian stimulation protocol, trigger	Oocytes retrieved, median (range)^2^	Oocytes cryopreserved, median (range)^◊^
					AMH ng/mL (n=52)	FSH mIU/mL (n=33)	AFC (n=25)			
Maxwell et al, USA (23)	TG male	Case Report	17	PM				Antagonist cycle	21	17
Rothenberg et al, USA (24)	TG male	Case Report	16	Pubertal suppression (GnRHa at 14 years, Tanner II)		0.89		GnRHa (never ceased), rFSH + hCG, -, hCG trigger	5	4
Wallace et al, USA (25)	TG male	Case Report	17	PM	3.5	5.7	40	rFSH + hMG, antagonist, GnRHa and hCG trigger	39	35
Martin et al, USA (26)	TG males	Case Report	15	Pubertal suppression (GnRHa at 10 years, Tanner II)	2.62	3.6	25	GnRHa, letrozole. rFSH + HMG, antagonist, hCG trigger	36	22
Insogna et al, USA (27)	TG males	Case Series (n=3)	15 (15-17)	PM (case 1 used GnRHa at 12)	3.1 (0.9-5.29)			hMG, hCG trigger/rFSH + hMG, antagonist, GnRHa trigger/rFSH + hMG, antagonist, HcG or GnRHa trigger	20 (15-31)	12 (10-18)
Chen et al, USA (28)	TG males	Case Series (n=5)	16 (14-18)	PM	5.9 (3.6-6.5)			rFSH ± hMG, antagonist, hCG trigger	19 (11-28)	13 (8-25)
Barrett et al, USA (29)	TG males	Case Series (n=17)	- (12-18)	-(case 2 - Pubertal suppression with GnRHa)	3.18 (0.44-12.87)	5.65 (1.7-9.5)		rFHS, hMG or both, GnRH antagonist protocol or low-dose down-regulation protocol with GnRHa, hCG or GnRHa trigger	22 (5-43)	14.5 (3-26)
Amir et al, Israel (30)	(i)TG males; (ii) Females with cancer	Retrospective Study (n=48)	- (13-18)	PM		TG male: mean 5.4 ± 1.7 Female: -	TG male: mean 19.8 ± 5.6	rFSH, antagonist, GnRHa or hCG or dual triggers	TG male: mean 30.6 ± 12.8 female: mean 22 ± 13.2	TG male: mean 25.6 ± 12.9 female: mean 18.8 ± 11.2
Reichman et al, USA (31)	MDS	Case Report	13	Peripubertal, Premenarcheal	0.95	5.0	9	hMG, antagonist. hCG trigger	20	18
Peddie et al, UK (32)	MDS	Case Report	14	PM		7.1	17	rFSH + hMG, antagonist, hCG trigger	13	12
Cai, H et al, China (33)	MDS	Case Report	17	PM		3.27	7	rFSH, GnRHa trigger.	17	13
Tsampras et al, UK (34)	Aplastic anaemia, MDS	Prospective Study (n=2)	17, 17	–	7.34,8.01^^^		20,19	Gonadotropins, antagonist, hCG trigger. DuoStim	2 cycles: 21 (20-22)	2 cycles: 17 (13-21)
Garg et al, USA (35)	Hodgkin’s Lymphoma	Case Report	14	PM	0.4		11	rFSH + hMG, antagonist, hCG trigger	13	11
Kutteh et al, USA (36)	Medulloblastoma	Case Series (n=3)	14 (13-15)	PM	3.61 (1.96-3.83)^^^		25 (15-31)	hMG, antagonist, GnRHa trigger	25 (18-26)	17 (12-23)
Kim at al, USA (37)	Pulmonary Hypertension	Case Report	17	–				Long agonist protocol rFSH and hMG	14	14
Lavery et al, UK (19)	Sickle Cell Disease	Case Series (n=8)	16 (14-18)	PM	10.6 (7.1-10.7)^^^ n=5 missing	4.25 (1.2-7.6)	16 (6-20)	rFSH. Combination of agonist and antagonist protocols. HCG or GnRHa trigger	10.5 (5-31)	9 (1-30)
Oktay et al, USA (38)	TS mosaicism	Case Report	14	PM	0.9, 1.7	5.3		rFSH, antagonist, GnRHa trigger/rFSH + hMG, antagonist, GnRHa trigger	2 cycles: 9 (7-11)	2 cycles: 6 (4-8)
Azem et al, Israel (10)	TS mosaicism	Case Report	7	Prepubertal	1.13	5.2	5, 3	rFSH & rLH. GnRHa trigger/rFSH + rLH. HCG trigger	2 cycles: 3 (0-6)	2 cycles: 3 (0-6)
Martel et al, USA (39)	TS or TS mosaicism, 47 XXX	Case Series (n=11)	15 (13-18)	Variation	1.04 (< 0.003-2.99) n=4 missing	36.65 (5.2-74) n= 7 missing		“gonadotropins”, antagonist, hCG ± GnRHa	1 cycle: 14 (0-21) 2 cycles: 0 (0-22) 3 cycles: 9.5 (0-19) 9 cycles: 3	1 cycle: 12 (0-16) 2 cycles: 0 (0-16) 3 cycles: 4 (0-8) 9 cycles: 0
Oktay et al, USA (40)	TS mosaicism, malignancy	Retrospective Study (n=4)^+^	13.5 (13-15)	PM	1.3 (0.76-1.6)	5.65 (5.6-7.8)	6 (5-11)	rFSH/hFSH + hMG or rLH. Antagonist, rHCG/hHCG/GnRHa triggers.	17.5 (8-21)	8 (4-10)
Hipp et al, USA (41)	Variety of diagnoses*	Retrospective Study (n=306)	- (≤18)	PM				In all age groups the most common stimulation protocol was antagonist		
Rodriguez-Wallberg et al, Sweden (42)	Variety of diagnoses**	Prospective Study (n=24)	16 (14-17)	PM				Not described	–	
Manuel, et al, USA (43)	Variety of diagnoses***	Retrospective Study (n=26)	16.5 (13-18)	–	2.72 (0.25-6.50)			rFSH +/- hMG, antagonist, hCG trigger.	13 (4-31)	10 (0-25)
Totals			15.2 (7-18)		2.9 (0.003-12.9)	4.5 (<0.1-20.5)	16 (5-35).		17 (0-43)	10 (0-35)

PM, post menarcheal; FSH, follicle stimulating hormone; AMH, anti mullerian hormone; AFC, antral follicle count; TG, transgender; MDS, myelodysplastic syndrome; rFSH, recombinant human follicle-stimulating hormone; GnRHa, gonadotropin releasing hormone agonist; rHCG, recombinant human chorionic gonadotropin; hMG, human menopausal gonadotropin; HCG, human chorionic gonadotropin; rLH, recombinant LH.

- = not described.

^#^Denominator changes due to missing data.

^◊^ 1 cycle unless otherwise specified.

^^^ AMH was converted to ng/mL for consistency.

^+^ One case was duplicate, described in Oktay, et al. (38).

* cancer, aplastic anaemia, sickle cell disease, autoimmune disease, gender dysphoria, unexplained infertility, TS, mosaic TS, diminished ovarian reserve, medical reasons not otherwise specified.

** cancer, TS, gender dysphoria, galactosemia, impending ovarian failure, benign ovarian, autoimmune disease, benign haematological, neurological disease.

***cancer, beta thalasaemia, aplastic anaemia, paroxysmal nocturnal haemoglobinuria, gender dysphoria, TS, panhypopituitarism, NMDA autoimmune encephalitis, multiple sclerosis, benign dermoid cyst.

The studies included 468 participants who underwent OS (median age 15.2 years, range 7-18) with a total of 488 cycles of OS completed. All but 18 of these cycles (96.3%) successfully resulted in mature oocyte cryopreservation.

### 3.2 Outcomes according to age

The four large cohort studies in this review (30, 41–43) described a total of 404 participants ≤ 18 years as grouped data, and did not provide a breakdown of outcomes in relation to age category or Tanner stage. Across the remaining 19 studies, 64 participants with a median age of 15 years (range 7-18) were described (**Table 1**).

There were three case reports of OS in premenarcheal children, one of whom was prepubertal (10). The prepubertal patient was a 7-year-old with mosaic TS (45,X[37]/47,XXX[15]) who initially underwent OS with gonadotropin-releasing hormone agonist (GnRHa) trigger which failed to yield oocytes. The second cycle with hCG trigger was successful, resulting in the retrieval of six oocytes and cryopreservation of all six mature oocytes. Martel et al. (39) described a 14-year-old premenarchal patient with TS who froze two oocytes over one cycle, using an hCG trigger. Her pubertal status was unknown. Reichman et al. (31) described a 13 year old premenarchal peri-pubertal (Tanner 3 breast and Tanner 1 pubic hair development) with myelodysplastic syndrome. An hCG trigger was used for this patient, and 18 mature oocytes were cryopreserved in one cycle, before gonadotoxic treatment commenced.

Another notably young patient ≤ 12 years-old (their exact age was not specified) was a transgender male who had 9 mature oocytes cryopreserved over 2 cycles (29). A further 31 patients aged 13-15 years old (10, 19, 26–29, 31, 32, 35, 36, 38–40) cryopreserved a median of 9.5 oocytes (range 0-22) and 31 patients aged 16-18 years old (19, 23–25, 27–29, 33, 34, 37, 39) cryopreserved a median of 14 oocytes (range 0-35).

A multi-center cohort study that assessed OS in oncology and non-oncology populations, compared outcomes in those aged 13 to 17 years with those aged 18 to 21 years (43). They reported that younger participants required higher doses of gonadotropins [median 2325IU FSH (range 0-3375) versus 2038IU (range 525-5850)] and froze fewer oocytes [median 11 (range 1-24) versus 13 (2-27)]. These differences were not, however, statistically significant. A retrospective study demonstrated that younger cohorts were also more likely to have cycles cancelled because of poor response (10% in those under 20-years, compared to 4.9%, 4.7% and 7.4% in the 20-29 year, 30-34 years and ≥35 year age groups respectively) (41). For those that proceeded, however, it was concluded that OS cycles in adolescent women were similar with regard to stimulation characteristics and oocyte yield to those in other age groups.

### 3.3 Outcomes according to clinical diagnosis

The four large cohort studies provided grouped data on diagnoses, which included cancer, haemoglobinopathies, aplastic anaemia, paroxysmal nocturnal haemoglobinuria, gender dysphoria, TS, panhypopituitarism, N-methyl D-aspartate (NMDA) autoimmune encephalitis, multiple sclerosis, benign dermoid cyst, galactosemia and unspecified (30, 41–43) (**Table 1**). For the remaining 19 studies with 64 participants, there were 29 patients who were transgender (23–29), 15 patients with a sex chromosome disorder (10, 38, 39), 10 patients with a cancer diagnosis (31–36, 40), one patient with aplastic anaemia (34), one patient with pulmonary hypertension (37) and one patient with sickle cell disease (19).

Those with TS or TS mosaicism cryopreserved a mean of 3.4 mature oocytes (range 0-16) (10, 38–40), compared with a mean of 12.3 mature oocytes (range 1-23) in all extractable cancer diagnoses (30–36) and 14.7 mature oocytes (range 3-35) in transgender males (24–30). One study described eight patients with Sickle Cell Disease who had a median of nine oocytes cryopreserved (range 1-30) (19) and another study described one patient with pulmonary hypertension who had 14 oocytes cryopreserved (37). Across all studies, five patients were not successful in retrieving any oocytes, over a total of 17 cycles (39). All these patients were diagnosed with either TS or mosaic TS.

Four transgender (TG) males described in four different case studies had treatment with GnRHa to suppress puberty prior to fertility preservation. In three of these patients the mean duration of GnRHa use was 3 years (range 2-5), and in the other patient the duration of use was not described. One study described a 16-year-old who commenced GnRHa therapy at 14 years of age, at Tanner stage 2 but menarcheal status not described, who continued this throughout the period in which the oocytes were obtained and cryopreserved: four mature oocytes were cryopreserved (24). Another case report (26) described a 15-year-old who had been on treatment to suppress puberty since the age of 10. This patient had their GnRHa implant removed prior to OS and an aromatase inhibitor was used to maintain low oestrogen concentrations during OS. Despite this, 22 mature oocytes were cryopreserved from one OS cycle. In another study a 15 year-old, who had puberty suppressed since 12 years old, continued GnRHa throughout the stimulation and retrieval, and had 12 oocytes cryopreserved from one cycle (27). One patient in a recent case series used GnRHa to suppress puberty and successfully cryopreserved 25 oocytes after one OS cycle (29). It is not clear in this study if this patient continued with the GnRHa suppression throughout the OS and at what pubertal stage this was commenced.

The only study directly comparing two cohorts with different diagnoses compared nine adolescent transgender males who had not used GnRHa, with 39 adolescent females with a cancer diagnosis. There was no significant difference in the mean age between the two groups (16.4 vs 15.5 years, respectively, *P = 0.064*). There was no difference in the mean number of days of FSH stimulation between them, however the amount of FSH used was significantly lower and the peak oestradiol levels were significantly higher among the transgender males, compared with the females (3073 pg/ml vs 1269 pg/ml respectively *P = 0.*018). Despite this, there was no significant differences in the number of retrieved oocytes (30.6 ± 12.8 vs 22 ± 13.2, *P=0.091*), the number of mature oocytes (25.6 ± 12.9 vs 18.8 ± 11.2, *P=0.134*) and the maturity rates (81.5 ± 10.0% vs 85.4 ± 14.6%, *P=0.*261) of oocytes between the two groups respectively (30).

### 3.4 Outcomes according to ovarian reserve testing

Some form of ovarian reserve testing was performed prior to commencing ovarian stimulation in 19 studies, and these values were analysed where possible (**Table 1**), however, reporting of these results was incomplete. Out of 468 participants, anti-mullerian hormone (AMH, ng/mL) was described in 52 participants (10, 19, 25–29, 31, 34–36, 38–40, 43), follicular stimulating hormone (FSH, mIU/mL) in 33 participants (10, 19, 24–26, 29–33, 38–40) and antral follicle count (AFC) in 25 participants (10, 19, 25, 26, 31–36, 40). Median AMH was 2.9ng/mL (range 0.003-12.9), median FSH was 4.5mIU/L (range <0.1-20.5) and median AFC was 16 (range 5-35).

There were some ovarian reserve testing results that were outside of standard expected ranges. Four patients described in one study (39) had FSH < 1mIU/mL. These patients aged 14-18 years old, all had a diagnosis of TS or TS mosaicism and had a median of 9.5 (0-19) oocytes retrieved and a median of 5 (0-15) oocytes cryopreserved. One further case report demonstrated FSH < 1mIU/mL in a TG male on GnRHa for puberty suppression (24). This patient had five oocytes retrieved, of which four were cryopreserved in one OS cycle. There was only one patient described with FSH > 10mIU/mL (39). This 14-year-old with TS had FSH of 20.6mIU/mL and AMH 0.03ng/mL and no oocytes were retrieved over three cycles. A further 11 patients of varying ages with diagnoses including transgender males, cancer and TS or TS mosaicism, had AMH <1.1ng/mL. The median number of oocytes retrieved was 12.8 (0-33), and cryopreserved was 8.7 (0-21). Four patients aged 7-15 years old with either TS or TS mosaicism had AFC < 7 indicating low functional ovarian reserve (10, 19, 40). These patients had a mean number of 9.2 (range 0-19) oocytes retrieved, and 5.2 (range 0-9) oocytes cryopreserved over five cycles. A low AFC (<7) was observed in one 14-year-old with leukaemia however 21 oocytes were retrieved, and 10 were cryopreserved over one OS cycle (40).

Outcomes for ovarian reserve testing outside of expected ranges were correlated with oocytes retrieved and cryopreserved (**Table 2**).

**Table 2:** Description of studies with ovarian reserve testing results outside of expected range.

Study	Age and diagnosis	AMH (ng/mL)	FSH (mIU/mL)	AFC	Oocytes retrieved, cryopreserved
Rothenberg et al, USA (24)	16, TG male	–	0.89	–	5,4
Barrett et al, USA (29)	13-15, TG male 16-18, TG male 16-18, TG male	0.73 0.44 0.59	–	–	5,5/15,8 (2 cycles) 9,8 33,21
Reichman et al, USA (31)	13, myelodysplastic syndrome	0.95	5.0	9	20,18
Garg et al, USA (35)	14, Hodgkin’s Lymphoma	0.4	–	11	13,11
Lavery et al, UK (19)	15, Sickle Cell Anaemia	–	4.8	6	5,4
Oktay et al, USA (38)	14, TS mosaicism	0.9	5.3	–	11,8
Azem et al, Israel (10)	7, TS mosaicism	1.1	5.2	5/3 (2 cycles)	0,0/6,6 (2 cycles)
Martel et al, USA (39)	14, TS 14, TS 15, TS mosaicism 15, TS 16, TS mosaicism 18, TS mosaicism	<0.16 0.03 <0.003 Unknown 1.63 Unknown	0.4 20.6 1.8 0.2 0.5 <0.1	–	4,2 0,0 0,0 15,15 19,8 0,0
Oktay et al, USA (40)	13, TS mosaicism 13, TS 14, Leukemia	1.59 0.76 0.8	5.7 5.6 7.8	6 6 5	19,9 16,7 21,10

### 3.5 Outcomes according to stimulation protocol

All protocols except those described in five studies were random start antagonist cycles that used recombinant FSH +/- human menopausal gonadotrophin for ovarian stimulation (**Table 1**) (10, 19, 24, 33, 37). One study described the failure of a GnRHa trigger to produce oocytes in a prepubertal child with TS, with subsequent success with hCG trigger in a second cycle (10). All other studies with premenarchal patients or those using GnRHa for pubertal suppression had successful oocyte retrieval following an hCG trigger (24, 26, 27, 31, 39). The remaining post pubertal patients, not on treatment to suppress puberty, had a combination of hCG and GnRHa trigger (19, 23, 25, 27–30, 32, 33, 35, 36, 38, 43). A 15-year-old transgender male (26) also commenced aromatase inhibitor (letrozole) during OS to maintain low oestrogen concentrations. Medication doses varied depending on individual protocols and patient characteristics and were therefore not comparable.

Two female patients ≤ 18 years old underwent a double ovarian stimulation (DuoStim) protocol for fertility preservation in one study (34). In these, a 17 year old with aplastic anaemia had nine oocytes cryopreserved in the first cycle and a further 12 oocytes cryopreserved in the second cycle with a five day interval between cycles. The other, a 17 year old with myelodysplasia had one oocyte cryopreserved in the first cycle, and a further 12 oocytes cryopreserved in the second cycle after a seven day interval. Treatment as planned prior to OS was not delayed and there were no reports of OHSS in either of these patients. In four studies (10, 29, 38, 39), more than one cycle was completed, which were either in transgender patients (2) or those who had a sex chromosome disorder (8).

Both transabdominal and transvaginal ultrasound were utilised throughout the studies to monitor follicular growth and maturation during stimulation. In one seven year old patient, transabdominal oocyte retrieval was performed, in which six oocytes were successfully retrieved (10). In 133 patients transvaginal retrieval was described (19, 26, 36, 38–40, 43), and in the remaining 334 participants retrieval method was not specified (23–25, 37, 41, 42).

### 3.6 Adverse outcomes

In all combined studies, complications were rare (<1%). The largest study was reported by Hipp et al. (41) which included 449 patients (of whom 306 were ≤ 18 years). Data on adverse outcomes was reported as group data, (comparing ages <20 years old with 20-29 years, 30-34 year and ≥ 35 years) and a more detailed sub-analysis in those ≤18 years was not available. They reported that there was a significantly increased risk of OHSS in those younger than 20 years of age (0.9%) compared to older women (0.4%). Other complications were also rare (<1%). In this study, in women <20 years, three women (0.67%) were either hospitalized or developed an infection. A further two patients described in two different studies (19, 29) experienced mild to moderate OHSS with one of these patients requiring three days of hospital admission for supportive treatment. In both these patients hCG was used to trigger oocyte maturation.

The mental burden due to treatment-related dysphoria in transgender males undergoing OS was also described in a 16-year-old transgender male who had vaginal bleeding for 7 days after oocyte retrieval and breast development. The patient reported depressed mood and brief passive suicidal thoughts in response to these symptoms (24), which regressed within 3 months.

No study commented on delays to cancer treatment or other therapy as a result of OS.

### 3.7 Pregnancy using cryopreserved oocytes

Only one study reported a pregnancy resulting in a live birth after long-term cryopreservation of oocytes, from a 17 year old female requiring gonadotoxic treatment for Pulmonary Hypertension (37). The oocytes were warmed after 5 years of storage and 2 embryos were transferred into a surrogate, due to the maternal medical condition, resulting in a healthy baby boy, delivered at term weighing 3,600g. No other patients have been reported to have utilised their frozen oocytes to create a pregnancy.

## 4 Discussion

Fertility preservation is very important to those requiring gonadotoxic treatments or those with medical conditions that impact future fertility, and as such is a rapidly expanding field. With advancements in cryopreservation methods over the past decade in the adult population, success rates with oocyte cryopreservation have improved significantly (44) but this approach remains poorly studied and understood in the paediatric and adolescent population.

This review included 468 participants who underwent a total of 488 OS cycles, with successful mature oocyte cryopreservation in all but 18 of these cycles (96.3%). An additional 53 cycles were cancelled for poor response (9.8%) however cancellation rates should be interpreted with caution due to the retrospective nature of the studies. This systematic review therefore demonstrates that OS and oocyte cryopreservation is achievable in the young although numbers remain small and long-term outcomes unknown. Of note,three studies broadly comparing the adolescent population with the adult population (41–43) reassuringly displayed no significant different number of oocyte cryopreserved between the different age cohorts. Outside of the larger cohort studies in this review, there was a trend to higher numbers of cryopreserved oocytes in the older age ranges [median 4.5 (range 0-6) in ≤ 12 years old, median 9.5 (range 0-22) in 13-15 year-olds, median 14 (range 0-35) in 16-18 year-olds]. The number of patients are however small.

Until recently, OS has only been described in post pubertal patients. There was only one patient in this study who was prepubertal and was successful in cryopreserving six oocytes. Another premenarchal patient with TS had a low yield of two mature oocytes. However, the third premenarchal patient, with a diagnosis of myelodysplastic syndrome, had 18 mature oocytes successfully cryopreserved in one cycle. This does challenge the traditional thinking that oocyte collection can only be considered in those who are physically and emotionally mature. But questions around the number and quality of such oocytes required to achieve parenthood remain unanswered. TS or TS mosaicism had a much lower rate of successful OS and oocyte retrieval with a mean of 3.4 mature oocytes frozen (range 0-16), compared with 12.3 (range 1-23) in all extractable cancer diagnoses and 14.7 (range 3-35) in transgender males. Patients with TS are known to have a greatly increased rate of oocyte depletion resulting in low or absent ovarian reserve (2, 10) and even where follicles are present, many of these follicles may show abnormalities that are likely to limit their potential to support fertility (45).

The data from this review show that the patients who had the greatest number of oocytes frozen per cycle were the transgender patients (25, 27, 29) and this included the four transgender males who had commenced GnRHa to suppress puberty prior to fertility preservation. All four of these patients were successful in cryopreserving mature oocytes although the number of oocytes varied from 4 to 25 and their pubertal and menarchal status were not always clear or available. Although these initial data are promising, more research is required to assess the impact of initiation of GnRHa for suppression of puberty, as well as ongoing gender-affirming hormone treatment, prior to and during OS cycles.

Regardless of diagnoses, there is a paucity of data regarding utility and pregnancy outcomes from oocyte cryopreservation in young patients and there is evidence that the prepubertal ovary contains significant numbers of follicles with abnormal morphology that seem to be lost during adolescence (17). Additionally, higher rates of fetal aneuploidy have been, higher rates of fetal aneuploidy have been described in adolescent pregnancy, when compared with women in their twenties (46). Therefore, the future ability to attain a viable pregnancy and live birth is uncertain, especially in the prepubertal cohort. Only a single case report of a 17 year old female who cryopreserved oocytes resulting in a successful pregnancy and livebirth (37) is described in the literature. Future studies should focus on prospective follow-up on long term reproductive outcomes, as well as assessing additional risk or long-term implications of stimulation of an immature Hypothalamic Pituitary Ovarian (HPO) axis.

The use of standard markers of ovarian reserve such as AMH and AFC in predicting response to OS in adolescents remains unclear (47). There is a discrepancy between unfavourable test results of ovarian reserve and the associated number of oocytes cryopreserved in some cases in this study. This is thought to be multifactorial in origin and could reflect differences in the stages of ovarian development at extremes of youth (19, 35). Reassuringly there were no examples of patients with normal ovarian reserve as indicated from testing, who then responded poorly to OS. In addition to markers of ovarian reserve, standardized monitoring and stimulation protocols in the paediatric and adolescent population are not well established and the variation in stimulation protocols amongst studies created challenges when comparing data. The only study in this review to use a DuoStim protocol (34) showed promising results with an increased number of oocytes retrieved in the second cycle, increasing the number of oocytes stored. Larger studies are required to establish appropriate assessment of ovarian reserve as well as designing optimum OS protocols in this population.

As the transvaginal ultrasound approach is often considered unacceptable in a young cohort, transabdominal ultrasound of the ovaries was frequently utilised for monitoring ovarian response to OS in the studies included in this review. Additionally, one study has described successful transabdominal oocyte retrieval in a prepubertal girl (10). The transabdominal approach of monitoring and retrieval is more technically challenging and superior visualization is generally achieved with a transvaginal probe in mature adults. It is therefore an important area of future research to assess the level of accuracy when monitoring ovarian reserve and successfully retrieving oocytes via a transabdominal approach.

It is essential to minimise the risk of harm from OS in the paediatric and adolescent population and consider the risks and benefits of this approach compared to ovarian tissue preservation (**Table 3**). This review demonstrates that the risk of OHSS exists, but appears to be no greater than in the adult, where the incidence of moderate and severe OHSS have been estimated to be 3-6% and 0.1-2% respectively (51). The absence of immediate embryo transfer contributes to this (52). Despite this, in the pre or peripubertal population with immature HPO axis, or the transgender populations where HCG trigger is often preferred, there is the potential for a higher risk of OHSS (53). In both cases of OHSS described in this review, where data about stimulation protocol were available, hCG was used as trigger (19, 29). Although the risks of OS and oocyte retrieval are not considered to be higher in those with TS, risk of death during pregnancy is increased by as much as 100-fold (54). Therefore, any patient who is deemed to have increased medical risk associated with carrying a pregnancy should be counselled about the option of surrogacy (55). Other medical conditions, such a sickle cell disease or cancer have a known predisposition to thrombosis and vasoocclusive events (56) underlying comorbidities which may affect safety during ovarian stimulation and ovarian response must be considered when assessing the value and mitigating risks of OS. Furthermore, it is known that the process of OS may be physically and emotionally demanding in an adult population, however the psychological impact in a young population is unknown. The risk of mental burden due to dysphoric triggers in transgender males undergoing OS is an important consideration as the process increases endogenous oestrogen production, may involve discontinuing or reducing the dose of testosterone or other gender affirming hormonal treatments, and the resumption of menses before beginning the process (57, 58).

**Table 3:** Pros and Cons of ovarian tissue cryopreservation compared to Oocyte Cryopreservation in those ≤ 18 years.

Ovarian tissue cryopreservation	Oocyte cryopreservation
Two reported pregnancies from prepubertal tissue, innovative procedure, now transitioned into standard practice (12, 13)	One pregnancy from post-pubertal oocyte collection. Consider experimental in prepubertal patients, innovative in post-pubertal patients under 18 years (37)
No delays to cancer therapy	Two-week delay to treatment, cannot be offered to those who require urgent cancer treatment
Can be done at any age	The youngest case report is 7 years of age (10)
No lengthy monitoring required for tissue harvest	Requires hormone treatment, monitoring with blood tests and scans which may cause morbidity in gender diverse and other populations
Requires careful selection to minimise morbidity	Requires careful selection to minimise morbidity
Minimally invasive surgery, low-risk procedure with careful patient selection (48)	Risks ovarian hyperstimulation syndrome <1%, and other complications<1%
May be undertaken as interval procedure after start of gonadotoxic therapy	Cannot be undertaken for at least 6 months after gonadotoxic therapy due to mutagenic risk (49)
Provides very high follicle density numbers: adult data suggests 25% chance of livebirth, success rates in the young are unknown (50)	Provides a finite number of oocytes: adult data suggests cumulative live births per patient 33.9-35.2% for women under 35 years, but success rates in the young are unknown (15)
Autotransplantation in gender diverse populations may not be tolerable to them. Carries a risk of malignant reseeding in some populations (3, 20)	Does not require auto-transplantation of tissue
Oocytes with abnormal morphology likely to undergo atresia	May theoretically increase yield of oocytes with abnormal morphology (17)

In those utilising OS for oocyte cryopreservation prior to cancer related therapies, current evidence does not suggest differences in survival and recurrence of cancer rates in adult patients who underwent OS prior to gonadotoxic cancer treatments compared with those who did not (59, 60) although this has yet to be studied in those 18 and younger. Furthermore OS is not considered a viable option in those in poor general condition who need to commence cancer treatment straight away, resulting in reportingc bias.

There were limitations in evaluating this review that may have impacted the analysis of outcomes. The description of ovarian reserve markers as well as baseline patient characteristics including BMI, Tanner stage and menstrual history was described in varying detail and often lacking amongst the studies. This could affect the comparison between patients and as such, results in this study should be interpreted with caution. Furthermore, discrepancies in monitoring and stimulation protocols amongst studies could impact the ability to compare overall outcomes.

The purpose in each study varied, with some studies comparing different diagnoses in their analysis and others comparing differing ages. Other studies reported broad outcomes for cohorts that included all ages from childhood to adulthood and encompassed a variety of diagnoses. Many of the larger studies in this review were not able to provide a breakdown of age in their description of results. The range of diagnoses and ages throughout the studies in this review may have significant impacts on the likelihood of success of COS making the results not necessarily applicable to alternate populations.

## 5 Conclusion

OS and oocyte cryopreservation is novel in the paediatric and adolescent population, but it offers hope to younger people and more diverse patient populations for the possibility of future biological parenthood. While it is considered standard practice in adults, long term outcomes are largely unknown in the young and the procedure should be considered experimental in prepubertal and premenarchal patients.

## Data availability statement

The original contributions presented in the study are included in the article/**Supplementary Material**. Further inquiries can be directed to the corresponding author.

## Author contributions

Conception and design: MS, MP, KM, DL, RA, KS, DG, YJ. Data Acquisition, Analysis: MS, KM. Data interpretation: MS, MP, KM, DL, RA, KS, DG, YJ. Critically reviewing drafts: MS, MP, KM, DL, RA, KS, DG, YJ. Critical review and approval of final draft: MS, MP, KM, DL, RA, KS, DG, YJ. Resources and supervision: MS, MP, YJ. All authors contributed to the article and approved the submitted version.

## Appendix 1

**Table 1:** Checklist for assessing the quality of quantitative studies.

Criteria		YES (2)	PARTIAL (1)	NO (0)	N/A
1	Question / objective sufficiently described?				
2	Study design evident and appropriate?				
3	Method of subject/comparison group selection or source of information/input variables described and appropriate?				
4	Subject (and comparison group, if applicable) characteristics sufficiently described?				
5	If interventional and random allocation was possible, was it described?				
6	If interventional and blinding of investigators was possible, was it reported?				
7	If interventional and blinding of subjects was possible, was it reported?				
8	Outcome and (if applicable) exposure measure(s) well defined and robust to measurement / misclassification bias? Means of assessment reported?				
9	Sample size appropriate?				
10	Analytic methods described/justified and appropriate?				
11	Some estimate of variance is reported for the main results?				
12	Controlled for confounding?				
13	Results reported in sufficient detail?				
14	Conclusions supported by the results?				

## Appendix 2

**Table 2:** Risk of bias assessment.

Author	1. Objective	2. Design	3. Subject selection	4. Subject characteristics	5. Random allocation	6. Blinding investigators	7. Blinding subjects	8. Outcomes	9. Sample size	10. Analysis	11. Estimate of variance	12. Cofounding	13. Results	14. Conclusion	Total Score (%)
Maxwell et al. (23)	No (0)	Yes (2)	Yes (2)	Partial (1)	N/A	N/A	N/A	Yes (2)	Yes (2)	No (0)	No (0)	No (0)	Partial (1)	Yes (2)	54%
Rothenberg et al. (24)	No (0)	Partial (1)	Yes (2)	Partial (1)	N/A	N/A	N/A	Yes (2)	Yes (2)	No (0)	No (0)	No (0)	Yes (2)	No (0)	45%
Wallace et al. (25)	No (0)	Yes (2)	Yes (2)	Yes (2)	N/A	N/A	N/A	Yes (2)	Yes (2)	Yes (2)	No (0)	No (0)	Yes (2)	Yes (2)	73%
Martin et al. (26)	Yes (2)	Yes (2)	Yes (2)	Yes (2)	N/A	N/A	N/A	Yes (2)	Yes (2)	Partial (1)	No (0)	No (0)	Yes (2)	Yes (2)	77%
Insogna et al. (27)	No (0)	Yes (2)	Yes (2)	Yes (2)	N/A	N/A	N/A	Yes (2)	Yes (2)	Partial (1)	No (0)	No (0)	Yes (2)	Partial (1)	64%
Chen et al. (28)	No (0)	Yes (2)	Yes (2)	Yes (2)	N/A	N/A	N/A	Yes (2)	Yes (2)	Partial (1)	No (0)	No (0)	Yes (2)	Partial (1)	64%
Barrett et al. (29)	Yes (2)	Yes (2)	Yes (2)	Yes (2)	N/A	N/A	N/A	Yes (2)	Yes (2)	Yes (2)	No (0)	No (0)	Yes (2)	Yes (2)	82%
Amir et al. (30)	Yes (2)	Yes (2)	Yes (2)	Yes (2)	N/A	N/A	N/A	Yes (2)	Yes (2)	Yes (2)	No (0)	Yes (2)	Yes (2)	Yes (2)	91%
Reichman et al. (31)	Yes (2)	Yes (2)	Yes (2)	Yes (2)	N/A	N/A	N/A	Yes (2)	Yes (2)	Partial (1)	No (0)	No (0)	Yes (2)	Partial (1)	73%
Peddie et al. (32)	Partial (1)	Yes (2)	Yes (2)	Yes (2)	N/A	N/A	N/A	Yes (2)	Yes (2)	Yes (2)	No (0)	No (0)	Yes (2)	Yes (2)	78%
Cai, H et al. (33)	No (0)	Yes (2)	Yes (2)	Yes (2)	N/A	N/A	N/A	Yes (2)	Yes (2)	Yes (2)	No (0)	No (0)	Yes (2)	Partial (1)	68%
Tsampras et al. (34)	No (0)	Yes (2)	Yes (2)	Partial (1)	N/A	N/A	N/A	Yes (2)	Yes (2)	Yes (2)	No (0)	No (0)	Yes (2)	Partial (1)	64%
Garg et al. (35)	No (0)	Yes (2)	Yes (2)	Yes (2)	N/A	N/A	N/A	Yes (2)	Yes (2)	Partial (1)	No (0)	No (0)	Yes (2)	Partial (1)	64%
Kutteh et al. (36)	No (0)	Yes (2)	Yes (2)	Yes (2)	N/A	N/A	N/A	Yes (2)	Yes (2)	Partial (1)	No (0)	No (0)	Yes (2)	Partial (1)	64%
Kim at al (37)	Yes (2)	Yes (2)	Yes (2)	Partial (1)	N/A	N/A	N/A	Yes (2)	Yes (2)	Partial (1)	No (0)	No (0)	Yes (2)	Partial (1)	68%
Lavery et al. (19)	Yes (2)	Yes (2)	Yes (2)	Yes (2)	N/A	N/A	N/A	Yes (2)	Yes (2)	Yes (2)	No (0)	No (0)	Yes (2)	Yes (2)	82%
Oktay et al. (38)	Yes (2)	Yes (2)	Yes (2)	Yes (2)	N/A	N/A	N/A	Yes (2)	Yes (2)	Partial (1)	No (0)	No (0)	Yes (2)	Partial (1)	73%
Azem et al. (10)	Yes (2)	Yes (2)	Yes (2)	Yes (2)	N/A	N/A	N/A	Yes (2)	Yes (2)	Yes (2)	No (0)	No (0)	Yes (2)	Yes (2)	82%
Martel et al. (39)	Yes (2)	Yes (2)	Yes (2)	Yes (2)	N/A	N/A	N/A	Yes (2)	Yes (2)	Yes (2)	No (0)	No (0)	Yes (2)	Yes (2)	82%
Oktay et al. (40)	Yes (2)	Yes (2)	Yes (2)	Yes (2)	N/A	N/A	N/A	Yes (2)	Yes (2)	Yes (2)	No (0)	No (0)	Yes (2)	Yes (2)	82%
Hipp et al. (41)	Yes (2)	Yes (2)	Yes (2)	Partial (1)	N/A	N/A	N/A	Yes (2)	Yes (2)	Yes (2)	Yes (2)	No (0)	Yes (2)	Yes (2)	86%
Rodriguez-Wallberg et al. (42)	Yes (2)	Yes (2)	Yes (2)	Partial (1)	N/A	N/A	N/A	Yes (2)	Yes (2)	Yes (2)	No (0)	No (0)	Yes (2)	Yes (2)	78%
Manuel, et al. (43)	Yes (2)	Yes (2)	Yes (2)	Partial (1)	N/A	N/A	N/A	Yes (2)	Yes (2)	Yes (2)	No (0)	Yes (2)	Yes (2)	Yes (2)	86%
