# Supplementary material for: Ovarian stimulation and oocyte cryopreservation in females and transgender males aged 18 years or less: a systematic review
Source: Front Endocrinol (Lausanne). 2023 Jun 19;14:1146476. doi: 10.3389/fendo.2023.1146476 (PMC10315913; doi:10.3389/fendo.2023.1146476)
Supplement: Supplementary file 1 [file DataSheet_1.docx]

**Supplementary Material**

1. **Literature Search**

Databases Last Searched: August 14, 2022

Database: Ovid MEDLINE

Search Strategy:

1. (ovulation induction or ovarian stimulation or oocyte retrieval).mp.
2. (adolescent/ or exp child/) not exp adult/
3. (adolescen* or young adult* or child* or teen* or peripubert* or peri-pubert* or prepuberty* or pre-pubert* or postpubert* or post-pubert* or girl*).ti.
4. 1 and (2 or 3)
5. ((ovarian stimulation or oocyte retrieval) adj5 (adolescen* or young adult* or child* or teen* or peripubert* or peri-pubert* or prepuberty* or pre-pubert* or postpubert* or post-pubert* or girl*)).tw,kw.
6. ((oocyte* or egg) adj4 (cryopreserve* or freezing) adj5 (adolescen* or young adult* or child* or teen* or peripubert* or peri-pubert* or prepuberty* or pre-pubert* or postpubert* or post-pubert* or girl*)).tw,kw.
7. 4 or 5 or 6
8. 7 not (animals/ not human/)

Database: Embase

Search Strategy:

1. Ovulation Induction/
2. (ovulation induction or ovarian stimulation or oocyte retrieval).mp.
3. 1 or 2
4. (adolescent/ or exp child/) not exp adult/
5. (adolescen* or young adult* or child* or teen* or peripubert* or peri-pubert* or prepuberty* or pre-pubert* or postpubert* or post-pubert* or girl*).ti.
6. 3 and (4 or 5)
7. ((ovarian stimulation or oocyte retrieval) adj5 (adolescen* or young adult* or child* or teen* or peripubert* or peri-pubert* or prepuberty* or pre-pubert* or postpubert* or post-pubert* or girl*)).tw,kw.
8. ((oocyte* or egg) adj4 (cryopreserve* or freezing) adj5 (adolescen* or young adult* or child* or teen* or peripubert* or peri-pubert* or prepuberty* or pre-pubert* or postpubert* or post-pubert* or girl*)).tw,kw.
9. 6 or 7 or 8
10. (conference abstract or conference paper or “conference review”).pt.
11. 9 not 10

Database: Cochrane Library

Search Strategy:

#1. MeSH descriptor: [Ovulation Induction] explode all trees

#2. (ovulation induction or ovarian stimulation or oocyte retrieval):ti,ab,kw

#3. #1 OR #2

#4. (adolescen* or young adult* or child* or teen* or peripubert* or peri-pubert* or prepuberty* or pre-pubert* or postpubert* or post-pubert* or girl):ti

#5. #3 AND #4

Database: Google Scholar

Search Strategy:

Oocyte cryopreservation|oocyte freezing|egg freezing|ovulation induction adolescent|young adult|children|child|teen|teenager|peripubertal|peri-pubertal or prepubertal|pre-pubertal|girl

1. **Supplementary Tables**

**Table S1 – Breakdown of age, ovarian reserve testing results and oocyte retrieval and cryopreservation**

| **Author, Country** | **Age in years**  **(mean, SD)** | **Ovarian reserve testing**  **(AMH ng/mL, FSH mIU/mL)** | **No. eggs retrieved, MII cryopreserved (patient**  **who has >1 cycle)** |
| --- | --- | --- | --- |
| Maxwell et al, USA (23) | 17 | - | 21,17 |
| Rothenberg et al, USA  (24) | 16 | FSH 0.89 | 5,4 |
| Wallace et al, USA (25) | 17 | AMH 3.5, FSH 5.7, AFC 40m | 39,35 |
| Martin et al, USA (26) | 15 | AMH 2.62, FSH 3.6, AFC 25 | 36,22 |
| Insogna et al, USA (27) | 15,15,17 | AMH 5.29 / 3.10 / 0.90 | 15,10 / 31,12 / 20,18 |
| Chen et al, USA (28) | 14,18,18,16,16 | AMH 6.5 / 5.9 / 5.9 / 3.6 / 4.3 | 11,8 / 19,14 / 13,13 / 20,11 / 28,25 |
| Barrett et al, USA (29) | 12-18 | AMH 1.58 / 1.53 / 3.10 / 3.26 / 0.73 / 2.7 /  4.64 / 3.27 / 9.84 / 3.00 / 3.60 / 12.87/  0.44 / 5.93 / 0.59 / 3.27  FSH median 5.65, range 1.7-9.5 | (13,3 / 9,6) / 26,5 / 15,9 / 22,16 / (5,5 / 15,8) /  25,20 / 19,14 / 22,15 / 32,18 / 26,19 / 22,18 /  43,14 / 9,8 / 21,17 / 33,21/ 30,26 |
| Amir et al, Israel (30) | 13-18 (TG 16.4  +/- 1.1,  cisgender F 15.5  +/- 1.3) | TG: mean FSH 5.4 ± 1.7, AFC 19.8 ± 5.6  Cisgender female: - | TG M: mean 30.6 ± 12.8, 25.6 ± 12.9  Cisgender F: mean 22 ± 13.2, 18.8 ± 11.2 |
| Reichman et al, USA  (31) | 13 | AMH 0.95, FSH 5.0, AFC 9 | 20,18 |
| Peddie et al, UK (32) | 14 | FSH 7.1, AFC 17 | 13,12 |
| Cai, H et al, China (33) | 17 | FSH 3.27, AFC 7 | 17,13 |
| Tsampras et al, UK (34) | 17, 17 | AMH* 7.34 / 8.01  AFC 20 / 19 | (9,9 / 13,12) / (1,1 / 19,12) |
| Garg et al, USA (35) | 14 | AMH 0.4, AFC 11 | 13,11 |
| Kutteh et al, USA (36) | 13,15,14 | AMH* 3.83 / 1.96 / 3.61  AFC 25 / 15 / 31 | 25,23 / 18, 12 / 26, 17 |
| Kim at al, USA (37) | 17 | - | 14,14 |
| Lavery et al, UK (19) | 14,15,16,16,16,17  ,18,18 | AMH* -/-/-/-/ 10.6 / 10.7 /-/ 7.1  FSH 2 / 4.8 / 1.2 / 4.3 / 7.6 / 4.2 / 7.6 / 2.9  AFC 13 / 6 / 18 / 16 / 16 / 20 / 20 / 12 | 7,7 / 5,4/ 21,16 / 29,25 / 14,11 / 5,3 / 31,30 /  7,1 |
| Oktay et al, USA (38) | 14 | AMH 0.9, 1.7  FSH 5.3 | 11,8 / 7,4 |
| Azem et al, Israel (10) | 7 | AMH 1.13, FSH 5.2, AFC 5, 3 | 0,0 / 6,6 |
| Martel et al, USA (39) | 13,14,14,14,15,15  ,15,16,16,17,18 | AMH 2.99 / <0.16 / 2.08 / 0.03 /-/-/ <  0.003 / 1.63 / 1.04 /-/- FSH  5.2/0.4/4.5/20.6/3.9/0.2/1.8/0.5/3.3/1.1/  <0.1 | 14,12 (1 cycle) / 4,2 (1 cycle) / 21,16 (1 cycle) /  0,0 (3 cycles) / 0,0 (2 cycles) / 15, 15 (1 cycle) /  0,0 (1 cycle) / 19,8 (3 cycles) / 22, 16 (2 cycles)  / 3, 0 (9 cycles) / 0, 0 (2 cycles) |
| Oktay et al, USA (40) | 13, 13, 14, 15 | AMH 1.59 / 0.76 / 1.6 / 0.8, 1.3  FSH 5.7 / 5.6 / 5.6 / 7.8  AFC 6 / 6 / 11 / 5 | 19, 9 +1 (IVM) / 16, 7 + 5 (IVM) / 8,4 / 21, 10 +  1 (IVM) |
| Hipp et al, USA (41) | ≤18 | - | Mean 18 oocytes retrieved in <20 year olds.  82.5% percent mature. |
| Rodriguez-Wallberg et  al, Sweden (42) | 14-17 (16.0) | - | - |
| Manuel, et al, USA (43) | 13-18 | AMH median 2.72 (0.25-6.50) | Median 13 (4-31) ,10 (0-25) |
